# Analysis of Th17-associated cytokines and clinical correlations in patients with dry eye disease

**DOI:** 10.1371/journal.pone.0173301

**Published:** 2017-04-05

**Authors:** Rongjun Liu, Caifeng Gao, Huijin Chen, Yaxin Li, Ying Jin, Hong Qi

**Affiliations:** 1 Department of Ophthalmology, Peking University Third Hospital, Beijing, China; 2 Guangdong Women and Children Hospital, Guangzhou, China; 3 The First Hospital of Fangshan District, Beijing, China; Ocular Surface Center, UNITED STATES

## Abstract

We aimed to investigate the expressions of three Th17-associated cytokines, interleukin (IL)-17A, IL-6 and IL-23, in protein and mRNA levels and their correlations with ocular surface parameters in patients with dry eye disease (DED) through a small sample size case control study. A total of 45 female subjects were divided into Sjögren’s syndrome (SS) DED group, non-Sjögren’s syndrome (non-SS) DED group and control group. Ocular surface disease index (OSDI) was self-answered and clinical tests including tear-film breakup time (BUT), Schirmer I test, cornea fluorescein staining (CFS) were performed. The conjunctival mRNA expressions of these cytokines were investigated by real-time polymerase chain reaction (PCR) and the levels of protein in tears were measured by mutiplex bead analysis. The correlations between cytokines and ocular surface parameters were analyzed. Results show that the expressions of IL-17A and IL-6 in protein and mRNA levels were both significantly increased in the DED group (P<0.05), and also higher in SS group comparing to the non-SS group (P<0.05). Moreover, IL-17A and IL-6 correlated well with ocular surface parameters (all P<0.05, R values range from 0.5–0.8). Despite the expression of IL-23 was significantly increased in the DED group (P<0.05), there was no significant difference found between the expressions of IL-23 in SS group and non-SS group (P>0.05) and no correlation found between the IL-23 and any ocular surface parameter (P>0.05). These findings indicates that the three Th17-associated cytokines, IL-17A, IL-6 and IL-23, play roles in the pathogenesis of DED and the expressions of IL-17A and IL-6 in tears have potential to be diagnostic biomarkers for DED.

## Introduction

Dry eye disease (DED) is an important public health problem characterized by increased osmolarity of the tear film and inflammation of the ocular surface which significantly hinders the performance of daily life and even may lead to blindness in severe cases[1, 2]. According to the report by Dry Eye Workshop (DEWS) in 2007, DED has two major classes, aqueous tear-deficient DED and evaporative DED. Aqueous tear-deficient DED also can be sub-classified into non-Sjögren’s (non-SS) DED and Sjögren’s syndrome (SS) DED[3]. SS is a autoimmune disease characterized by lymphocytic infiltration of the exocrine glands and dry eye is a common symptom[4]. Increasing evidence suggests that all DED are characterized by varying ocular surface inflammation[5, 6]. T-cell infiltration of the ocular surface is a pervasive finding in DED. It is now recognized that CD4^+ ^T cells including T helper 1 (Th1) cells and T helper 17 (Th17) cells are thought to be the effector T cells and moreover, Th17 cells are primarily responsible for maintaining the chronic and relapsing course of the disease[7, 8]. Several studies have shown that increased levels of inflammatory cytokines such as interleukin (IL)-1, IL-6, IL-8 and tumor necrosis factor (TNF)-α found in tears[9–12], and Tan et al showed that IL-17 and IL-22, the effective cytokines of Th17 cells, significantly increased in tears of patients with DED[13]. Thus, inflammatory cytokines particularly Th17-associated cytokines may play roles in the pathogenesis of the disease.

Nowadays, there are a large number of clinical investigative tests for the evaluation of dry eye. The options include tear-film breakup time (BUT) measurement, Schirmer I test, cornea fluorescein staining (CFS), tear osmolarity tests[14] and ocular surface disease index (OSDI). However, none of the available tests are considered to be the gold standard[15]. Therefore, it is significant to find a potential diagnostic biomarker for DED. Some studies analyzed the correlation between the expressions of inflammatory cytokines in protein levels and ocular surface parameters of patients with DED[10, 16, 17]. However, few study analyzed the expressions of Th17-associated cytokines in both protein and mRNA levels systematically and comprehensively. We detected the mRNA expressions of 84 Th17-associated cytokines in conjunctival epithelium cells by polymerase chain reaction (PCR) array. The result showed that the conjunctival mRNA expressions of IL-17A, IL-6 and IL-23 in patients with DED were particularly increased comparing to control group[18]. To further evaluate their roles in the pathogenesis of DED, especially the Th17 mediated mechanism, we not only investigated the expressions of the three Th17 associated cytokines in both protein and mRNA levels, but also analyzed their correlations with ocular surface parameters in this study to determine which cytokines have potential to be possible diagnostic biomarkers and therapeutic targets for DED.

## Materials and methods

### Patient selection

This study was approved by the Biomedical Ethics Committee of Peking University. All the subjects were postmenopausal women aged from 49 to 79 years and recruited from the Department of Ophthalmology, Peking University Third Hospital between 2013 and 2014. Written informed consent was obtained from each participants enrolled in this study. All the samples collected from the subjects were numbered from 1 to 45 in order to identify individual subjects and their data.

DED patients were divided into two groups: SS group and non-SS group. The inclusion and criteria for DED were as follows: patients should have the dry eye symptoms (>3 months), high OSDI scores (>13 points), abnormal tear-film BUT<10 seconds, low Schirmer I test (<10 mm), and positive CFS[19]. The CFS score is evaluated using 12 points scoring system[20]. Patients with DED in SS group should meet the diagnosis of primary SS criteria proposed by the American-European Consensus group[21]: dry mouth for more than 3 months, positive histopathology outcomes for salivary glands, objective evidence of salivary-gland involvement, presence in the serum of antibodies to Ro(SSA) or La(SSB) antigens, or both. Other patients were brought into non-SS group. Subjects in the control group were evaluated with all of the above measures and demonstrated that OSDI scores≤13 points, tear-film BUT≥10 seconds, Schirmer I test≥10 mm and negative CFS. The exclusion criteria were as follows: an infection or inflammatory disease not associated with dry eye; ocular surgical history with in the last 3 months; ocular therapies other than artificial tears; systemic diseases such as diabetes and hypertension; Meibomian gland dysfunction (MGD) over grade 1, the grade is according to the report of the International Workshop on MGD in 2011[22].

Tear samples and conjunctival epithelial tissues were collected in November 2013 to February 2014, and the study conducted in March 2014.

### Tear sample collection

Tear collection was performed before any other test and with a maximum of 10 minutes after the patient answered the OSDI questionnaire. Tear samples were collected non-traumatically from the inferior tear meniscus of both eyes. Care was taken to avoid additional tear reflex as much as possible. Glass capillary micropipettes (Drummond Scientific, Broomall, PA) were used to collect 1 μl of tears. Tear samples from two eyes (1 μl total) were fully eluted into a sterile collection tube containing 9 μl of 0.1% bovine serum albumin (Sigma-Aldrich, St. Louis, MO). Tubes with tear samples were kept cold (4°C) during collection, and sealed with a cap containing a rubber O-ring to prevent evaporation then stored at -80°C until activity assays were performed.

### Mutiplex bead analysis

The levels of IL-17A, IL-6 and IL-23 in tears of patients were measured using a Milliplex Map Kit (Human Th17 Magnetic Bead Panel, Millipore, Billerica, MA). The reactions were detected with the Bio-Plex Luminex 200 XYP instrument (Bio-Rad Laboratories). The sensitivity of the IL-17A, IL-6 and IL-23 concentrations was >0.7, 0.7 and 0.7 pg/mL, respectively.

### Conjunctival impression cytology

Impression cytology was performed to get the conjunctiva epithelial cells. A piece of nitrocellulose membrane (4mm × 5mm× 7mm × 10mm) (Pall Corp, America) were pressed lightly against the nasal and temporal bulbar conjunctiva after instillation of topical anesthesia (0.5%tetracaine hydrochloride, Alcon China Ophthalmic Product Company Ltd, Beijing, China). The 4 pieces of membrane from both two eyes were placed in a 1.5ml tube which contained 350μl cell lysis buffer (Qiagen, #74004). Tubes were stored at -80°C until further examination. RNA was extracted as using an RNA easy Microkit (Qiagen, #74004).

### Real-time PCR

The first-strand cDNA was synthesized from 0.4 μg of total RNA using the PrimeScript^™^ RT reagent kit with gDNA Eraser (TaKaRa, Dalian, China). Real-time PCR was performed using SYBR Premix Ex Taq system (TaKaRa, Dalian, China) and ABI PRISM 7500 detection system (Applied Biosystems) according to the manufacturer’s recommendations. Assays were performed in duplicate. A non-template control was included in all the experiments to evaluate DNA contamination of the reagent used. The primers used in this study (TaKaRa, Dalian, China) are listed in Table 1. The results of quantitative real-time PCR were analyzed by the comparative threshold (CT) cycle method and normalized by GAPDH as an internal control.

**Table 1:** Primer sequences used for real-time PCR.

Gene	Accession No.	Labeled Forward Primer	Unlabeled Reverse Primer
IL-6	NM_000600	AAGCCAGAGCTGTGCAGATGAGTA	TGTCCTGCAGCCACTGGTTC
IL-17A	NM_002190	ACCTGAACATCCATAACCGGAATAC	AGCGTTGATGCAGCCCAAG
IL-23	NM_016584	GAACAACTGAGGGAACCAAACC	GAATCTCTGCCCACTTCCACTT
GAPDH	NM_001256799	GCACCGTCAAGGCTGAGAAC	TGGTGAAGACGCCAGTGGA

### Statistical analysis

Statistical analyses were performed using SPSS for Windows version 19.0 software (SPSS Inc. Chicago, Illinois, USA). For all measurements, results from each group were expressed as mean ± SD. Differences among the 3 groups were tested using an ANOVA test, post hoc tests were performed by bonferroni. Correlations between the expressions of cytokines and ocular surface parameters (OSDI, BUT, Schirmer I test and CFS) were analyzed by Spearman correlation coefficient, respectively. P<0.05 is considered as statistically significant.

## Results

### Demographic and ocular surface parameters data

A total of 30 patients with DED (fifteen in SS group and fifteen in non-SS group) were included. Fifteen healthy volunteers constituted the control group. The demographic data and results of ocular surface parameters are presented in Table 2. There were no significant differences in mean age among the three groups (P = 0.473, P>0.05). Overall, there were statistically significant differences among the three groups in OSDI score, Schirmer I Test, BUT and CFS score (all P<0.05). The comparison results showed that OSDI score and CFS score of patients in SS group were both higher than the non-SS group (P = 0.02, P<0.001), while Schirmer I Test and BUT values were lower than the non-SS group (P = 0.048, P<0.001); The OSDI score and CFS score of patients in non-SS group were significantly higher than control group (all P<0.001), and Schirmer I Test, BUT values were significantly lower than control group (all P<0.001).

**Table 2:** Demographics and results of ocular surface parameters of patients.

	Control Group (n = 15)	DED Group	
		Non-SS (n = 15)	SS(n = 15)
Age(year)	64.37± 8.34	64.00± 11.00	66.75± 9.38^#^
OSDI Score	3.37 ± 3.30	42.63 ± 17.00*	51.43 ± 19.03+*
Schirmer I Test (mm)	15.47± 7.17	3.50± 2.25*	1.97 ± 1.55+*
BUT(s)	10.23 ± 0.61	3.60 ± 1.08*	1.89 ± 1.45+*
CFS	0	5.16 ± 1.65*	8.77 ± 2.84+*

DED, Dry Eye Disease; Non-SS, non-Sjögren’s syndrome; SS, Sjögren’s syndrome; OSDI, ocular surface disease index; BUT, break up time; CFS, cornea fluorescent staining.

^#^P>0.05 among three group

*P <0.05 compared with control group

^**+**^P <0.05 compared with Non-SS group

### Tear cytokines concentrations

**Fig 1:**
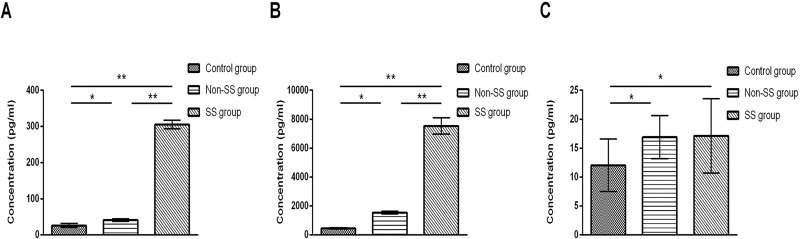
The levels of IL-17A, IL-6 and IL-23 in tears of control group and DED patients in SS and non-SS group. Results represent the mean ± SD. The P values were determined with ANOVA test. SS, Sjögren’s syndrome; non-SS, non- Sjögren’s syndrome.*, P < .05; **, P < .01.

The mean concentration of IL-17A in tears was 454.67 ± 37.70 pg/mL in control group, 1545.26 ± 91.95 pg/mL in non-SS group and 7539.50 ± 568.36 pg/mL in SS group. The mean concentration of IL-6 in tears was 26.25 ± 5.20 pg/mL in control group, 41.14 ± 3.52 pg/mL in non-SS group and 305.21 ± 12.14 pg/mL in SS group. The mean concentration of IL-23 in tears was 12.06 ± 4.53 pg/mL in control group, 16.91 ± 3.72 pg/mL in non-SS group and 17.15 ± 6.35 pg/mL in SS group. The levels of IL-17A, IL-6 and IL-23 in tears were significantly increased in non-SS group (P = 0.042, P = 0.03, P<0.001) and SS group (P<0.001, P<0.001, P<0.001) compared with control group. The levels of IL-17A and IL-6 in tears were significantly increased in SS group (P<0.001, P<0.001) compared to non-SS group (Fig 1). However, there was no significant difference found between the levels of IL-23 in SS group and non-SS group (P = 0.712).

### mRNA expression analysis of cytokines in conjunctiva

**Fig 2:**
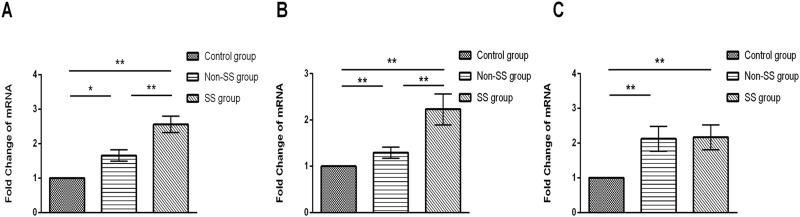
The mRNA expressions of IL-17A, IL-6 and IL-23 in conjunctival epithelial cells of control group and DED patients in SS and non-SS group. Results represent the mean ± SD. The P values were determined with ANOVA test. SS, Sjögren’s syndrome; non-SS, non- Sjögren’s syndrome. *, P< .05; **, P < .01.

Results of the real-time PCR for IL-17A, IL-6 and IL-23 are described in Fig 2. Compared with the control group, patients in non-SS group (P = 0.002, P = 0.11, P = 0.001) and SS group (P<0.001, P<0.001, P = 0.001) showed a significant increase in the mRNA expressions of IL-17A, IL-6 and IL-23. Moreover, patients in SS group (P<0.001, P = 0.002) showed a more significant increase in the mRNA expressions of IL-17A and IL-6 than patients in non-SS group. On the other hand, patients in SS group did not show a significant increase or decrease in the mRNA expression of IL-23 compared with the patients in non-SS group (P = 0.802).

### Correlations between cytokines and ocular surface parameters in patients with DED

**Fig 3:**
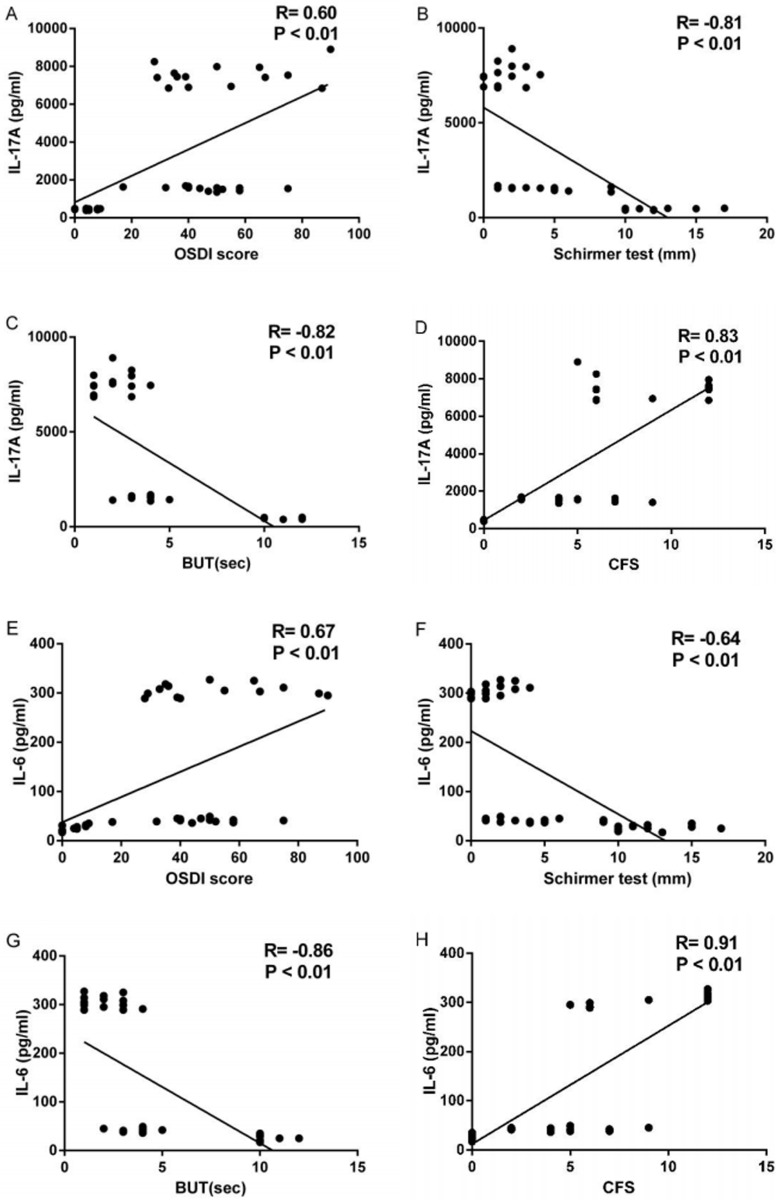
Correlation between levels of IL-17A and IL-6 in tears and ocular surface parameters including OSDI score (A, E), Schirmer I Test (B, F), BUT (C, G), and CFS (D, H). The R and P values were determined with Spearman correlation coefficient. IL, interleukin; OSDI, ocular surface disease index; BUT, break up time; CFS, corneal fluorescent staining.

**Fig 4:**
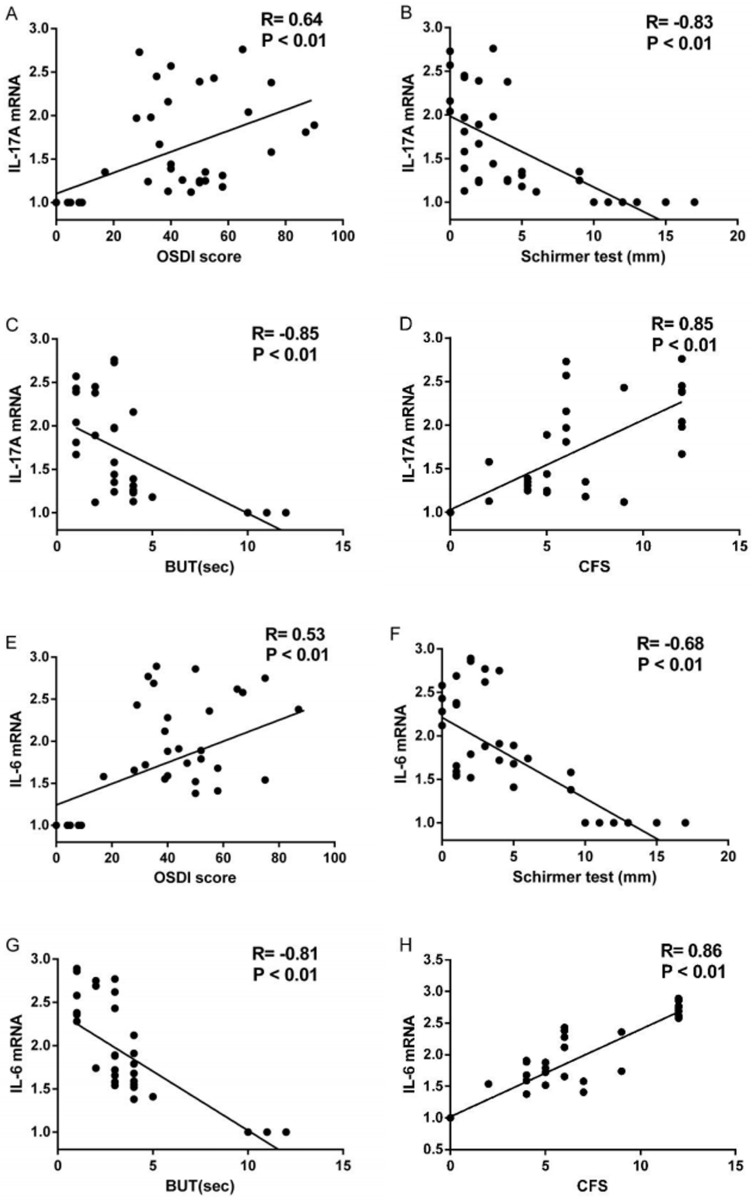
Correlation between conjunctival mRNA expressions of IL-17A and IL-6 and ocular surface parameters including OSDI score (A, E), Schirmer I Test (B, F), BUT (C, G), and CFS (D, H). The R and P values were determined with Spearman correlation coefficient.IL, interleukin; OSDI, ocular surface disease index; BUT, break up time; CFS, corneal fluorescent staining.

The correlations between the expressions of IL-17A, IL-6 and IL-23 and ocular surfaces parameters of 45 subjects were evaluated both in protein and mRNA levels. The correlation analysis between the expression of IL-23 in protein and mRNA levels and any ocular surface parameter showed no statistically significant (all P>0.05). On the other hand, the levels of IL-17A in tears correlated well with OSDI score (R = 0.60, P<0.001; Fig 3A), Schirmer I test (R = -0.81, P<0.001; Fig 3B), BUT (R = -0.82, P<0.001, Fig 3C) and CFS score (R = 0.83, P<0.001; Fig 3D).The IL-17A mRNA expression in conjunctiva correlated well with OSDI score (R = 0.64, P<0.001; Fig 4A), Schirmer I test (R = -0.83, P<0.001; Fig 4B), BUT (R = -0.85, P<0.001; Fig 4C) and CFS score (R = 0.85, P<0.001; Fig 4D). The levels of IL-6 in tears correlated well with OSDI score (R = 0.67, P<0.001; Fig 3E), Schirmer I test (R = -0.64, P<0.001; Fig 3F), BUT (R = -0.86, P<0.001; Fig 3G) and CFS score (R = 0.91, P<0.001; Fig 3H). The IL-6 mRNA expression in conjunctiva correlated well with OSDI score (R = 0.53, P<0.001; Fig 4E), Schirmer I test (R = -0.68, P<0.001; Fig 4F), BUT (R = -0.81, P<0.001; Fig 4G) and CFS score (R = 0.86, P<0.001; Fig 4H).

## Discussion

DED is a type of ocular surface inflammation where signs and clinical symptoms do not always correlate well[23–25]. Also, the diagnosis of DED is hampered by the lack of precise objective tests. Thus, it is significant to find possible diagnostic biomarkers for DED. Previous studies have been done in order to investigate cytokines to be biomarkers of DED. Studies by Kang et al[26] and Oh et al[11] showed that the concentration of IL-17 was significantly elevated in tear and serum from DED patients. Zheng et al[27] found that the expression of IL-23 was elevated in DED mice model under desiccating environmental stress. Findings of Yoon et al[10] and Lam et al[17]showed that the level of IL-17 in tears from DED patients was increased. The correlation studies revealed associations between some of inflammatory mediators such as IL-6, IL-17 and IL-22 and ocular surface parameters[12, 13, 17]. However, few study specifically focused on Th17-associated cytokines and limited data have been published evaluating the expressions of cytokines in mRNA levels.

Our results are almost consistent with previous studies. It demonstrated that the expressions of IL-17A, IL-6 and IL-23 on the ocular surfaces of DED patients significantly increased in both protein and mRNA levels. Thus, the increasing levels of IL-17A, IL-6 and IL-23 in tears are not the result of evaporative effects, but of overproduction. Besides, the expressions of IL-17A and IL-6 in both protein and mRNA levels were significantly increased in SS group comparing to the non-SS group. The combination of transforming growth factor-β (TGF-β) and IL-6 promotes the initial differentiation of naive CD4^+^ T cells to IL-17-producing Th17 cells, while subsequent exposure to IL-23 is required for the functional maturation and pathogenic function of Th17 cells[28, 29]. IL-17A secreted by Th17 cells finally infiltrates the tissues and lead to the inflammation[30]. Therefore, IL-17A, IL-6 and IL-23 might play the same roles in the pathogenesis of DED. IL-6 and IL-23 are the inducing factors of Th17 cells, while IL-17A is the effect factor of Th17 cells.

In addition, our study showed that the expression of IL-17A correlated well with ocular surface parameters in both protein and mRNA levels, same as the IL-6. Besides, analysis results of tears concentrations are almost paralleled with the results of conjunctival mRNA expressions. The R values range from 0.53–0.91, which means a moderate to high correlation existing. The expressions of IL-6 and IL-17A moderately correlated with OSDI score (R values range from 0.5–0.8), and there were some patients had relatively low expressions of IL-6 and IL-17A, while OSDI scores are pretty high, it might result from the different subjective symptom of patients with DED. While expressions of IL-6 and IL-17A highly correlated with Schirmer I Test, BUT and CFS (R values over 0.8). This is a primary study to investigate the correlation between the cytokines and clinical parameters. However, whether it is linear or non-linear correlations still needed further investigated. Since it is noninvasive and convenient to collect tears, the levels of IL-17A and IL-6 in tears have potential to be possible diagnostic biomarkers for DED.

However, as for the conjunctival mRNA expression and tears concentration of IL-23, no difference was found between patients in SS group and non-SS group. Additionally, there was no correlation found between the expression of IL-23 in protein and mRNA levels and any ocular surface parameter. IL-23 is essential for the full and sustained differentiation of Th17 cells[31]. The major source of IL-23 is different types of antigen presenting cells such as activated dendritic cells, monocytes and macrophages[32]. IL-23 upregulates the expression of IL-23 receptor in Th17 cells directly or indirectly through TGF-β and promotes the expansion of Th17 cells through its receptor and signaling pathway. Thus, exposure to a certain amount of IL-23 can cause the induction of functionally mature pathogenic Th17 cell[33, 34] and the concentration of IL-23 in tears is independent of the function of IL-23 in the Th17 mediated pathogenesis of DED.

There are certainly limitations in our study. Only 45 subjects involved in this study and all subjects were female. In addition, the dry eye patients have not been divided into different groups based on severity.

In summary, these results suggested that the expressions of IL-17A, IL-6 and IL-23 on ocular surfaces of DED patients significantly increased in both protein and mRNA levels. IL-17A, IL-6 and IL-23 play roles in the pathogenesis of DED. The expressions of IL-17A and IL-6 on ocular surface were higher in SS group and also correlated well with ocular surface parameters of DED. The levels of IL-17A and IL-6 in tears have potential to be the diagnostic biomarkers and therapeutic targets for DED. However, since DED is a very complex disease, further studies are needed to determine the exact role of Th17-associated cytokines, as well as their mutual link, in disease pathogenesis.

## Supporting information

S1 File Approval document in English. (DOCX)

S2 File Approval document in Chinese. (TIF)

S3 File Clinical studies checklist. (DOCX)

S4 File STROBE checklist. (DOCX)

S5 File Dataset. (RAR)
