# Supplementary material for: Analysis of Th17-associated cytokines and clinical correlations in patients with dry eye disease
Source: PLoS One. 2017 Apr 5;12(4):e0173301. doi: 10.1371/journal.pone.0173301 (PMC5381865; doi:10.1371/journal.pone.0173301)
Supplement: S1 File — (DOCX) [file pone.0173301.s001.docx]

**Biomedical ethics committee of Peking University**

**Ethics approval document**

| Project name：Analysis of Th17-associated cytokines in patients with ocular surface diseases | | |
| --- | --- | --- |
| Principle investigator：Hong Qi | Technical post：Associate chief physician | Mobile phone：13901066899 |
| Name of unit：Peking university third hospital | | |
| Project source：□Government □Foundation □Company □International organization  □independence √others | | |
| Research funding donors：The scientific Research Foundation for the Excellent Returned Overseas Chinese Scholars, Peking University Third Hospital | | |
| Type of review：√New project □Project after revised □Tracking review | | |
| Opinion from ethical review：  The research program and informed consent of the study —analysis of Th17-associated cytokines in patients with ocular surface diseases was reviewed by ethics committee, the result is that:  √meeting the requirements and approved  □research program or□informed consent will be approved after □revising or □ supplying information，  □review after revising  □disapproved  Biomedical ethics committee of Peking University  Signature （chairman of committee）：  Ruicong Peng | | |
