# Supplementary figures and images for: Analysis of Th17-associated cytokines and clinical correlations in patients with dry eye disease
Source: PLoS One. 2017 Apr 5;12(4):e0173301. doi: 10.1371/journal.pone.0173301 (PMC5381865; doi:10.1371/journal.pone.0173301)

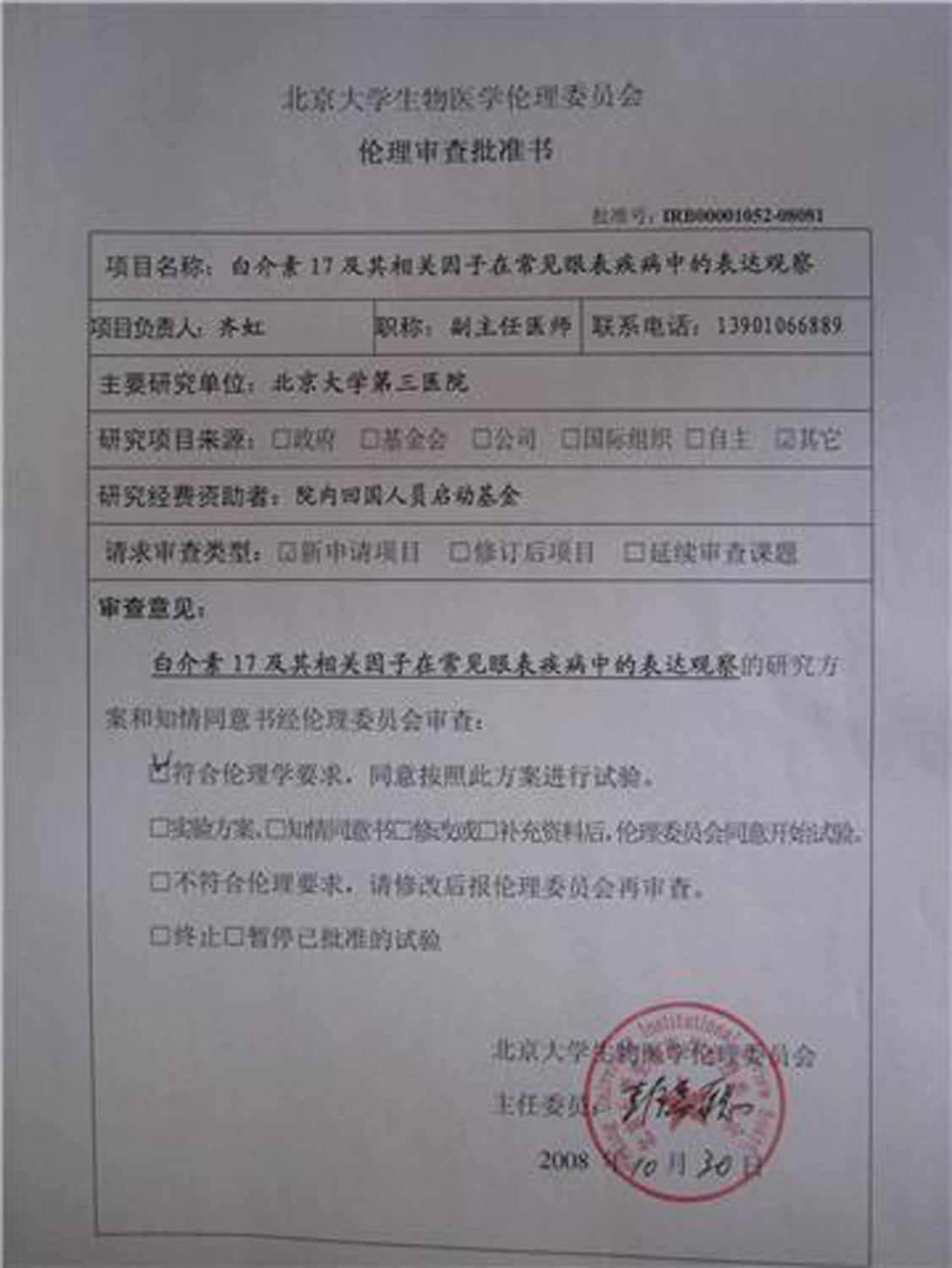

Supplement: S2 File — (TIF) [file pone.0173301.s002.tif]
